# Unmasking the Ulcer: Colonic Pyogenic Granuloma

**DOI:** 10.51894/001c.166041

**Published:** 2026-08-01

**Authors:** Joann P. Wongvravit, Brandon Travis Wiggins, Jeffrey Sobecki, Mark Rigby, Mark Joseph Minaudo

**Affiliations:** 1 Henry Ford - Genesys

## 133

### Introduction

Colonic pyogenic granuloma or lobular capillary hemangioma, is a rare, benign vascular lesion forming throughout the gastrointestinal (GI) tract, usually asymptomatic but can lead to anemia and occult GI bleed. The incidence rate is currently unknown as these tumors are rare and typically occur on the skin and oral mucosa. Our case demonstrates a single superficial ulceration discovered on routine colonoscopy.

### Case Presentation

A 68-year-old female presented for a colonoscopy after an episode of diverticulitis. She was asymptomatic after completion of antibiotics. Her colonoscopy revealed a 5 mm transverse colon polyp, sigmoid colon stenosis, and focal colitis with a superficial ulceration (Figure 1). The ulcer was initially suspected to be related to subclinical diverticulitis. However, pathology revealed polypoid tissue with lobular arrangement of capillaries and classic epithelial collarette, consistent with pyogenic granuloma (Figure 2). The patient did well after the procedure and no further management was necessary.

### Discussion

Pyogenic granulomas (PG) are composed of lobular arrangements of capillaries with the same histopathology as lesions found on the skin and oral mucosa (1,3,4). To the best of our knowledge, there have been less than 200 cases reporting PG in the GI tract (3,4). Of these studies, two larger series of 23 cases and 34 cases reported the colorectum as the most common site for PG, specifically the sigmoid colon (3,4). The pathogenesis is not clearly understood, however one-third of PG are believed to develop secondary to mucosal trauma followed by medications, chronic irritation, and hormonal imbalances (3,4). In addition, PG in the GI tract are likely overlooked as contributing to occult GI bleed and symptomatic anemia as these lesions are rare (1,2). Interestingly, the colonoscopy in our case was performed two months post-resolution of sigmoid diverticulitis which may have predisposed our patient to mucosal injury leading to pyogenic granuloma formation. Therefore, there should be a high degree of clinical suspicion for GI pyogenic granulomas after mucosal inflammation or trauma, especially if associated with anemia and/or occult GI bleed. Further studies are required to investigate appropriate management and follow-up strategies for these lesions due to the rarity of GI pyogenic granulomas.

